# Supplementary material for: Anti-Inflammatory and Neuroprotective Effects of Undaria pinnatifida Fucoidan
Source: Mar Drugs. 2025 Aug 29;23(9):350. doi: 10.3390/md23090350 (PMC12471492; doi:10.3390/md23090350)
Supplement: Supplementary file 1 [file marinedrugs-23-00350-s001.zip › marinedrugs-3818904-supplementary.pdf]

Supplementary Materials

Table S1. Study-level characteristics of *Undaria pinnatifida* fucoidan (UPF) used in Sections 3-4.

Reference No. in main text	Compound source	Supplier	Extraction/ purification	Molecular weight (kDa)	Sulphation (%)	Uronic acid (%)	Purity	Monosaccharide composition (%)	Reference
[22]	UPF	Marinova Pty Ltd. (Tasmania, Australia)	NR	NR	28.3%	NR	89.3%	Fuc (22.5%), Xyl (0.3%), Gal (19%), Ara (0.6%), Rha (0.6%)	[1]
[25]	UPF	Bright Moon Seaweed Group Co., Ltd. (Qingdao, China)	NR	141.7 kDa	29.9%	>1.0%	90%	Fuc: Gal: Man: GlcA (92.7: 33.1: 1.5: 1.0)	[2]
[26]	Crude fucoidan from sporophyll of *Undaria pinnatifida*	Golfo Nuevo (Northern Patagonia, Argentina)	Acid (HCL) extraction	NR	21.9%	NR	NR	Fuc (52.4%), Gal (41.7%), Rha (2.5%), Glu (1.4%), Ara (1%), Xyl (1%), Man (1%)	[3]
[27]	UPF	Marinova Pty Ltd. (Tasmania, Australia)	NR	5–30 kDa	NR	NR	≥90%	NR	[4]
[28]	Fucoidan from sporophyll of *Undaria pinnatifida*	Heawon Co., Inc. Republic of Korea	NR	5–30 kDa	NR	NR	NR	NR	[5]
[30]	Fucoidan from *Undaria pinnatifida* Harvey	Sigma-Aldrich (Spain)	NR	>80 kDa	38.44%	NR	NR	Fuc (27.1%), Gal (24.78%)	[6]

[31]	Fucoidan from sporophyll of *Undaria pinnatifida*	Haerim Fucoidan Co., Ltd. (Wando, Republic of Korea)	NR	258.7 kDa	30.9%	10.9%	NR	Fuc (21%), Gal (23%), Man (0.9%)	[7]
[33]	UPF	Local seafood market (China)	Ethanol extraction	428 kDa	9.55%	7.19%	NR	Man: Rha: GlcA: Glu: Gal: Ara: Fuc (32.1: 2.9: 7.1: 14.1: 22.3: 3.3: 17.92)	[8]
[34]	Fucoidan from *Undaria pinnatifida* Harvey	Haewon Biotech, Inc. Republic of Korea	NR	NR	NR	NR	NR	NR	[9]
[35]	Extract from sporophyll of *Undaria pinnatifida*	Local market in Wando (Jeollanam-do, Korea)	Ultrasonic extraction and ethanol precipitation	447–1062 kDa	NR	NR	27.5–80.2%	Fuc (29.4–32.3%), Rha (0.3%), Gal (34.9–59.3%), Glu (4.5–21.9%), Xyl (2.9–9.0%), Fru (2.5%)	[10]
[40]	Extract from sporophyll of *Undaria pinnatifida*	Sigma–Aldrich (St Louis, MO, USA)	Ultrasonic extraction	390–767 kDa	9.8–15.5%	NR	NR	NR	[11]
[42]	Sulphated *Undaria pinnatifida* polysaccharide	Shaanxi Ciyuan Biotechnology Co., Ltd.	Dialysis and ethanol precipitation	8.3–10.2 kDa	1.5–36.3%	NR	NR	Fuc (51%), Gal (48%	[12]
[43]	Fucoidan from sporophyll of *Undaria pinnatifida*	Heawon Co, Inc (Seoul, South Korea)	NR	NR	25%	NR	NR	NR	[13]
[44]	UPF	Algamar (Pontevedra, Spain)	Microwave-assisted extraction	23.6 and 58 kDa	1.7%	NR	NR	Glu (2.7%), Gal+Xyl+Man (10.6%), Rha (0.9%), Fuc (11.4%)	[14]

[45]	UPF	Marinova Pty Ltd. (Tasmania, Australia)	Aqueous extraction	NR	29.9%	4.6%	86.9%	Fuc (44.1%), Xyl (0.8%), Man (4.3%), Gal (45.1%), Glu (2.9%), Ara (1.6%), Rha (1.2%)	[15]
[46]	Lyophilised UPF	Sigma-Aldrich (St Louis, USA)	NR	NR	NR	NR	NR	NR	[16]
[47]	UPF	Sigma-Aldrich (St Louis, USA)	NR	54 kDa	NR	NR	NR	NR	[17]
[48]	Fucoidan from sporophyll of *Undaria pinnatifida*	Haewon Biotech Co., Ltd. (Seoul, Korea)	NR	NR	31.7%	NR	NR	Fuc (64.4%), Gal (31.9%), Man (1.3%)	[18]
[49]	UPF	Marinova Pty Ltd. (Tasmania, Australia)	NR	NR	28.3%	NR	89.3%	Fuc (22.5%), Xyl (0.3%), Gal (19%), Ara (0.6%), Rha (0.6%)	[19]
[50]	Sulphated *Undaria pinnatifida* polysaccharide	Changxing Commercial Market in Dalian (Liaoning, China)	Ethanol extraction	425–750 kDa	21.5%	6.1%	NR	Man: Rha: GalA: Glu: Gal: Xyl: Fuc (22.7: 6.1: 1.1: 8.2: 29.8: 5.1: 26.7)	[20]
[51]	UPF	NR	Ethanol extraction	NR	NR	NR	NR	NR	[21]
[57]	UPF	Dalian Haibao Biotechnology Co., Ltd. (Liaoning, China)	NR	NR	NR	NR	NR	NR	[22]
[58]	*Undaria pinnatifida* powder	Changdao (Yantai, China)	NR	NR	NR	NR	NR	NR	[23]

[59]	Sulphated *Undaria pinnatifida* polysaccharide	Local market in Dalian (Liaoning, China)	Ethanol extraction	425–750 kDa	NR	NR	>92%	Man: Rha: GalA: Glu: Gal: Xyl: Fuc (22.7: 6.1: 1.1: 8.2: 29.8: 5.1: 26.7)	[24]
[60]	UPF	Marinova Pty Ltd. (Tasmania, Australia)	NR	NR	28.3%	NR	89.3%	Fuc (22.5%), Xyl (0.3%), Gal (19%), Ara (0.6%), Rha (0.6%)	[25]
[61]	UPF	Local seafood market in Dalian (Liaoning, China)	NR	428 kDa	5.5%	NR	NR	Man: Rha: GlcA, Glu: Gal: Ara: Fuc (32.1: 2.9: 7.2: 14.2: 22.3: 3.3: 17.9)	[26]
[67]	UPF	Qingdao Brightmoon Seaweed Group Co.,Ltd. (Qingdao, China)	NR	260 kDa	32%	4%	NR	Fuc (55.5%), Gal (14.3%), Rha (7.0%)	[27]
[68]	Fucoidan from sporophyll of *Undaria pinnatifida*	Haerim Fucoidan Co. (Wando, Korea)	Water extraction	≥200 kDa	30%	NR	90%	Fuc (22%), Gal (25%), Man (2%)	[28]
[71]	*Undaria pinnatifida* powder	Gijang (South Korea)	Ethanol extraction	63–160 kDa	NR	NR	NR	NR	[29]
[72]	UPF	Qingdao Bright Moon Seaweed Group Co., Ltd. (Qingdao, China)	NR	290 kDa	18.6%	2.6%	>96%	Man: GlcA: Glu: Gal: Xyl: Fuc (3: 1: 1: 13.8: 1.3: 10.2)	[30]
[84]	UPF	Marinova Pty Ltd. (Tasmania, Australia)	NR	48–84 kDa	23.2–31%	0.9–5%	89.8–96%	Fuc (21.7–23.7%), Xyl (1.4–1.9%), Gal (16.1–21.4%), Glu (1.3%), Ara (0.8%), Rha (0.4%)	[31]

[85]	Crude fucoidan from sporophyll of *Undaria pinnatifida*	Dalian Aquaculture Group Co., Ltd. (Dalian, China)	Water extraction	266 kDa	12.6%	13.9%	>90%	NR	[32]
[86]	*Undaria pinnatifida* powder	NR	Ethanol extraction	NR	NR	NR	NR	NR	[33]
[88]	UPF	Sigma-Aldrich (USA, F8315)	NR	<10 kDa	NR	NR	>95%	NR	[34]
[90]	Commercial fucoidan	Sigma (St Louis, MO, USA)	NR	NR	NR	NR	NR	NR	[35]
[92]	*Undaria pinnatifida* powder	Gijang aquaculture farm (Busan, Korea)	Ethanol extraction	NR	NR	NR	NR	NR	[36]
[94]	Commercial fucoidan	Sigma (St Louis, MO, USA)	NR	NR	NR	NR	NR	NR	[37]
[95]	Commercial fucoidan	Sigma (St Louis, MO, USA)	NR	NR	NR	NR	NR	NR	[38]
[96]	Low-molecular-weight fucoidan	NR	NR	7 kDa	NR	NR	NR	NR	[39]
[97]	Fucoidan polysaccharide sulfate	Kangyue Biotech (Xian, China)	NR	NR	NR	NR	NR	NR	[40]

Fucose (Fuc), xylose (Xyl), galactose (Gal), arabinose (Ara), rhamnose (Rha), mannose (Man), glucuronic acid (GlcA), glucose (Glu), Fructose (Fru), galacturonic acid (GalA), non-report (NR), and *Undaria pinnatifida* fucoidan (UPF).

References

1. Yang, C., et al., *Neuroprotective and Anti-Inflammatory Activity of Undaria pinnatifida Fucoidan In Vivo—A Proteomic Investigation.* Marine Drugs, 2025. **23**(5): p. 189.
2. Shi, F.-S., et al., *Fucoidan from Ascophyllum nodosum and Undaria pinnatifida attenuate SARS-CoV-2 infection in vitro and in vivo by suppressing ACE2 and alleviating inflammation.* Carbohydrate Polymers, 2024. **332**: p. 121884.
3. Giuliani, M., et al., *Undaria pinnatifida fucoidan extract inhibits activation of the NF-κB signaling pathway by herpes simplex virus type 1 and prevents amyloid-β peptide synthesis in retinal pigment epithelium cells.* Archives of Virology, 2025. **170**(2): p. 27.
4. Ahmad, T., et al., *Anti-Inflammatory Activity of Fucoidan Extracts In Vitro.* Marine Drugs, 2021. **19**(12): p. 702.
5. Kim, K.-J., K.-Y. Yoon, and B.-Y. Lee, *Low molecular weight fucoidan from the sporophyll of Undaria pinnatifida suppresses inflammation by promoting the inhibition of mitogen-activated protein kinases and oxidative stress in RAW264.7 cells.* Fitoterapia, 2012. **83**(8): p. 1628-1635.
6. Vaamonde-García, C., et al., *Study of fucoidans as natural biomolecules for therapeutical applications in osteoarthritis.* Carbohydrate Polymers, 2021. **258**: p. 117692.
7. Lim, J.-M., H.J. Yoo, and K.-W. Lee, *High Molecular Weight Fucoidan Restores Intestinal Integrity by Regulating Inflammation and Tight Junction Loss Induced by Methylglyoxal-Derived Hydroimidazolone-1.* Marine Drugs, 2022. **20**(9): p. 580.
8. Zheng, W., et al., *Undaria pinnatifida fucoidan ameliorates dietary fiber deficiency-induced inflammation and lipid abnormality by modulating mucosal microbiota and protecting intestinal barrier integrity.* International Journal of Biological Macromolecules, 2023. **247**: p. 125724.
9. Phull, A.-R., et al., *In vitro and in vivo evaluation of anti-arthritic, antioxidant efficacy of fucoidan from Undaria pinnatifida (Harvey) Suringar.* International Journal of Biological Macromolecules, 2017. **97**: p. 468-480.
10. Lee, J.H., et al., *The Antioxidant Activity of Undaria pinnatifida Sporophyll Extract Obtained Using Ultrasonication: A Focus on Crude Polysaccharide Extraction Using Ethanol Precipitation.* Antioxidants (Basel), 2023. **12**(11).
11. Song, K.-M., et al., *High yield ultrasonication extraction method for Undaria pinnatifida sporophyll and its anti-inflammatory properties associated with AP-1 pathway suppression.* LWT - Food Science and Technology, 2015. **64**(2): p. 1315-1322.
12. Chen, X.-W., et al., *Sulfated Undaria pinnatifida polysaccharides inhibit kidney stone formation through crystalline modulation and relieving cellular oxidative damage and inflammation.* Biomaterials Science, 2025. **13**(6): p. 1512-1528.
13. Kim, K.-J. and B.-Y. Lee, *Fucoidan from the sporophyll of Undaria pinnatifida suppresses adipocyte differentiation by inhibition of inflammation-related cytokines in 3T3-L1 cells.* Nutrition Research, 2012. **32**(6): p. 439-447.
14. Vaamonde-García, C., et al., *In Vitro Study of the Therapeutic Potential of Brown Crude Fucoidans in Osteoarthritis Treatment.* International Journal of Molecular Sciences, 2022. **23**(22): p. 14236.
15. Wimmer, B.C., et al., *Undaria pinnatifida Fucoidan Enhances Gut Microbiome, Butyrate Production, and Exerts Anti-Inflammatory Effects in an In Vitro Short-Term SHIME® Coupled to a Caco-2/THP-1 Co-Culture Model.* Marine Drugs, 2025. **23**(6): p. 242.
16. Yang, J.-H., *Topical Application of Fucoidan Improves Atopic Dermatitis Symptoms in NC/Nga Mice.* Phytotherapy Research, 2012. **26**(12): p. 1898-1903.

17. Li, X., et al., *Fucoidan from Undaria pinnatifida prevents vascular dysfunction through PI3K/Akt/eNOS-dependent mechanisms in the l-NAME-induced hypertensive rat model.* Food & Function, 2016. **7**(5): p. 2398-2408.
18. Herath, K., et al., *The Role of Fucoidans Isolated from the Sporophylls of Undaria pinnatifida against Particulate-Matter-Induced Allergic Airway Inflammation: Evidence of the Attenuation of Oxidative Stress and Inflammatory Responses.* Molecules, 2020. **25**(12).
19. Shanmugasundaram, D., et al., *Fucoidan Ameliorates Testosterone-Induced Benign Prostatic Hyperplasia (BPH) in Rats.* Res Rep Urol, 2024. **16**: p. 283-297.
20. Jiang, P., et al., *Sulfated polysaccharides from Undaria pinnatifida improved high fat diet-induced metabolic syndrome, gut microbiota dysbiosis and inflammation in BALB/c mice.* International Journal of Biological Macromolecules, 2021. **167**: p. 1587-1597.
21. Kang, K.S., et al., *Undaria pinnatifida fucoidan extract protects against CCl4-induced oxidative stress.* Biotechnology and Bioprocess Engineering, 2008. **13**(2): p. 168-173.
22. Liu, M., et al., *Fucoidan alleviates dyslipidemia and modulates gut microbiota in high-fat diet-induced mice.* Journal of Functional Foods, 2018. **48**: p. 220-227.
23. Li, L., et al., *Undaria pinnatifida improves obesity-related outcomes in association with gut microbiota and metabolomics modulation in high-fat diet-fed mice.* Applied Microbiology and Biotechnology, 2020. **104**(23): p. 10217-10231.
24. Zhang, P., et al., *Polysaccharides from edible brown seaweed Undaria pinnatifida are effective against high-fat diet-induced obesity in mice through the modulation of intestinal microecology.* Food & Function, 2022. **13**(5): p. 2581-2593.
25. Yang, C., et al., *Fucoidan from Undaria pinnatifida Enhances Exercise Performance and Increases the Abundance of Beneficial Gut Bacteria in Mice.* Marine Drugs, 2024. **22**(11): p. 485.
26. Zheng, W., et al., *Undaria pinnatifida fucoidan contributes to anti-inflammation activity of Bacteroides in fiber-deficient mice via modulation of gut microbiota and protection of intestinal barrier integrity.* International Journal of Biological Macromolecules, 2023. **252**: p. 126256.
27. Ren, P., et al., *Fucoidan exerts antitumor effects by regulating gut microbiota and tryptophan metabolism.* International Journal of Biological Macromolecules, 2025. **300**: p. 140334.
28. Park, E.-J., et al., *High-molecular-weight Fucoidan exerts an immune-enhancing effect in RAW 264.7 cells and cyclophosphamide-induced immunosuppression rat by altering the gut microbiome.* International Immunopharmacology, 2024. **139**: p. 112677.
29. Yu, Z.N., et al., *Undaria pinnatifida extract attenuates combined allergic rhinitis and asthma syndrome by the modulation of epithelial cell dysfunction and oxidative stress.* Acta Biochim Biophys Sin (Shanghai), 2024. **57**(5): p. 792-804.
30. Men, Q., et al., *Fucoidan alleviates Salmonella-induced inflammation and mortality by modulating gut microbiota and metabolites, protecting intestinal barrier, and inhibiting NF-κB pathway.* Food Bioscience, 2023. **56**: p. 103209.
31. Alghazwi, M., et al., *Comparative study on neuroprotective activities of fucoidans from Fucus vesiculosus and Undaria pinnatifida.* International Journal of Biological Macromolecules, 2019. **122**: p. 255-264.
32. Wei, H., et al., *Protective Effects of Fucoidan on Aβ25-35 and d-Gal-Induced Neurotoxicity in PC12 Cells and d-Gal-Induced Cognitive Dysfunction in Mice.* Marine Drugs, 2017. **15**(3).
33. Kim, J., et al., *Algae Undaria pinnatifida Protects Hypothalamic Neurons against Endoplasmic Reticulum Stress through Akt/mTOR Signaling.* Molecules, 2015. **20**(12): p. 20998-1009.

34. Chen, M., et al., *Low molecular weight fucoidan induces M2 macrophage polarization to attenuate inflammation through activation of the AMPK/mTOR autophagy pathway.* European Journal of Pharmacology, 2025. **986**: p. 177134.
35. Jhamandas, J.H., et al., *Fucoidan inhibits cellular and neurotoxic effects of β-amyloid (Aβ) in rat cholinergic basal forebrain neurons.* European Journal of Neuroscience, 2005. **21**(10): p. 2649-2659.
36. Mohibbullah, M., et al., *Neuroprotective effects of fucoxanthin and its derivative fucoxanthinol from the phaeophyte Undaria pinnatifida attenuate oxidative stress in hippocampal neurons.* Journal of Applied Phycology, 2018. **30**(6): p. 3243-3252.
37. Hu, C., G. Zhang, and Y.-t. Zhao, *Fucoidan attenuates the existing allodynia and hyperalgesia in a rat model of neuropathic pain.* Neuroscience Letters, 2014. **571**: p. 66-71.
38. Che, N., Y. Ma, and Y. Xin, *Protective Role of Fucoidan in Cerebral Ischemia-Reperfusion Injury through Inhibition of MAPK Signaling Pathway.* Biomol Ther (Seoul), 2017. **25**(3): p. 272-278.
39. Wang, T., M. Zhu, and Z.Z. He, *Low-Molecular-Weight Fucoidan Attenuates Mitochondrial Dysfunction and Improves Neurological Outcome After Traumatic Brain Injury in Aged Mice: Involvement of Sirt3.* Cell Mol Neurobiol, 2016. **36**(8): p. 1257-1268.
40. Wang, X., K. Yi, and Y. Zhao, *Fucoidan inhibits amyloid-β-induced toxicity in transgenic Caenorhabditis elegans by reducing the accumulation of amyloid-β and decreasing the production of reactive oxygen species.* Food & Function, 2018. **9**(1): p. 552-560.
